# Magnetic Resonance Imaging of Autoimmune Demyelinating Diseases as a Diagnostic Challenge for Radiologists: Report of Two Cases and Literature Review

**DOI:** 10.3390/life12040488

**Published:** 2022-03-28

**Authors:** Antonio Pierro, Alessandro Posa, Tiziana Addona, Antonella Petrosino, Vittorio Galasso, Alessandro Tanzilli, Sara Niro, Fernando Antonio Simone, Savino Cilla, Roberto Iezzi

**Affiliations:** 1 Dipartimento di Radiologia, Ospedale Regionale, “A. Cardarelli”, ASReM, Contrada Tappino, 86100 Campobasso, Italy; antonio.pierro@asrem.org (A.P.); vittogala@virgilio.it (V.G.); nirosara@yahoo.it (S.N.); fernandosimone1967@libero.it (F.A.S.); 2 Dipartimento di Diagnostica per Immagini, Radioterapia Oncologica ed Ematologia, U.O.C. Radiologia Diagnostica e Interventistica Generale, Fondazione Policlinico Universitario, “A. Gemelli”—IRCCS, L.go A. Gemelli 8, 00168 Rome, Italy; antonella.petrosino1990@gmail.com (A.P.); alessandrotanzilli93@gmail.com (A.T.); roberto.iezzi@policlinicogemelli.it (R.I.); 3 Dipartimento di Neurologia, Ospedale Regionale, “A. Cardarelli”, ASReM, Contrada Tappino, 86100 Campobasso, Italy; tiziana.addona@asrem.org; 4 Unità di Fisica Medica, Ospedale Gemelli Molise, Università Cattolica del Sacro Cuore, Contrada Tappino, 86100 Campobasso, Italy; savino.cilla@gemellimolise.it

**Keywords:** demyelinating diseases, spinal cord, neuromyelitis optica, disseminated encephalomyelitis, multiple sclerosis

## Abstract

The magnetic resonance characteristics of autoimmune demyelinating diseases are complex and represent a challenge for the radiologist. In this study we presented two different cases of detected autoimmune demyelinating diseases: one case of acute disseminated encephalomyelitis and one case of neuromyelitis optica, respectively. Expected and unexpected findings of magnetic resonance imaging examination for autoimmune demyelinating diseases were reported in order to provide a valuable approach for diagnosis. In particular, we highlight, review and discuss the presence of several uncommon imaging findings which could lead to a misinterpretation. The integration of magnetic resonance imaging findings with clinical and laboratory data is necessary to provide a valuable diagnosis.

## 1. Introduction

Autoimmune demyelinating diseases (ADD) constitute a heterogeneous group of diseases with variable clinical and imaging manifestations [1]. Multiple sclerosis (MS), neuromyelitis optica spectrum disorder (NMOSD), acute disseminated encephalomyelitis (ADEM), myelin oligodendrocyte glycoprotein (MOG) encephalomyelitis, and idiopathic transverse myelitis (TM) represent the main diseases characterized by autoimmune demyelination [1,2]. ADD are chronic disorders that can manifest with severe and acute neurologic complications due to direct damage to brain tissue and spinal cord [3,4,5]. Among all ADD, the archetype is the MS, representing the most common variant.

Diagnosing ADD can be very challenging owing to their complex and variable radiological features. Magnetic resonance imaging (MRI) plays a pivotal role in the diagnostic workflow [6,7]. However, misdiagnosis of ADD remains a matter of concern [8,9]. A valuable diagnosis is therefore necessary to establish an appropriate therapy, as therapeutic strategies greatly differ between ADD pathologies, influencing the patient’s prognosis. In this scenario, the evaluation of MRI features represents a major challenge for the radiologist and, in some cases, it may be a source of confusion, as certain ADD can present overlapping radiological findings. Radiologist expertise in recognition of the typical signs of the various ADD phenotypes needs to be strengthened with the integration of clinical and laboratory findings.

Aim of this paper is to describe two cases of diagnosed ADD: a case of ADEM and a case of NMO, respectively, which posed a real diagnostic challenge due to their heterogeneous radiological presentation, atypical for ADD, and to review epidemiological, clinical, laboratory and imaging characteristics of this group of disorders.

## 2. Materials and Methods

### 2.1. Case Presentation 1

**Figure 1:**
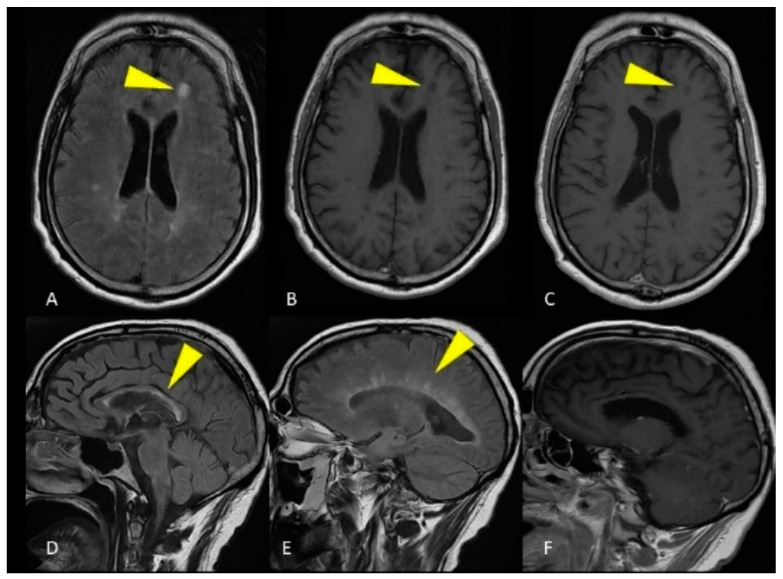
MRI of the brain. Axial FLAIR image (**A**) and unenhanced T1-weighted image (**B**) show a single ovoid-shaped lesion in the left frontal deep white matter, without enhancement on the T1-weighted post-gadolinium acquisition (**C**). Sagittal FLAIR image shows hyperintense lesions affecting the ependymal surface of the corpus callosum with a marble pattern (**D**). The so-called Dawson fingers are visible on the sagittal FLAIR image as hyperintense, ovoid lesions perpendicular to the body of the lateral ventricle (**E**). Corpus callosum and periventricular lesions don’t show enhancement on the T1-weighted post-gadolinium acquisition (**F**).

**Figure 2:**
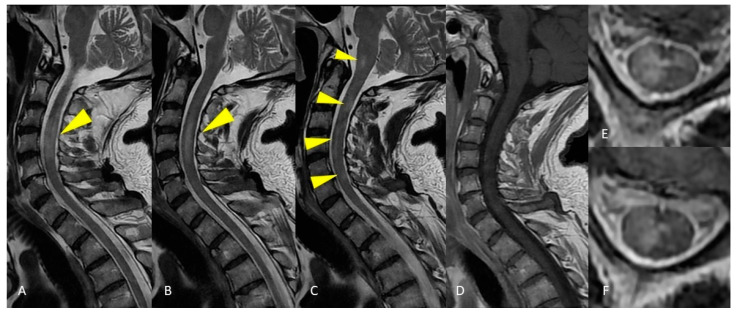
MRI of the cervical spinal cord. Sagittal T2-weighted images of the cervical spine show areas of patchy and long-segment (>1.5 vertebral body length) hyperintensity (**A**–**C**) without enhancement on the T1-weighted post-gadolinium acquisition (**D**). Axial T2-weighted image shows large hyperintensity involving half or more than half of the cross-sectional area of the spinal cord (**E**,**F**).

**Figure 3:**
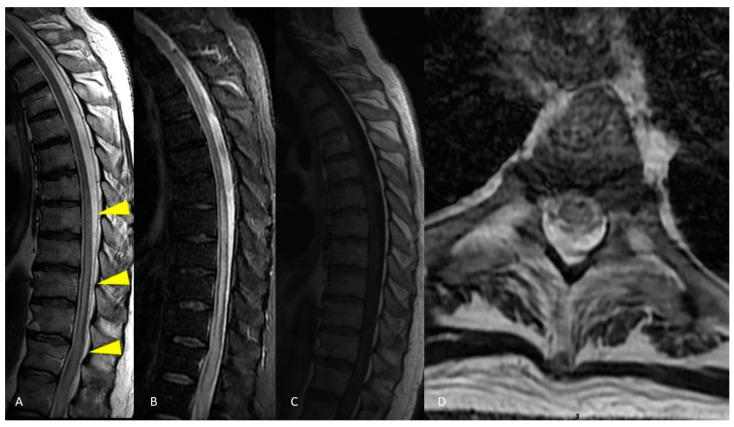
MRI of the thoracic spinal cord. Sagittal T2-weighted images demonstrate long segment hyperintensity of the thoracic cord without expansion or enhancement (**A**–**C**). Axial T2-weighted image shows large hyperintensity affecting all the cross-sectional area of the spinal cord (**D**).

A 57-year-old male with a history of chronic alcoholism presented to the emergency department with severe muscle weakness, which started with a progressive impairment in walking, followed by a feverish state (38 Celsius degrees), worsening up to being unable to walk. The patient was alert, collaborating, oriented in space, disoriented in time. Due to this clinical presentation, the patient was transferred to the neurology department for appropriate treatment. Osteo-tendon reflexes were normal in the upper limbs but absent in the lower limbs. Autoimmune screening, blood chemistry tests, and neoplastic markers were within normal limits. The search for pathogenic microorganisms was negative. Cyanocobalamin serum levels were normal. Anti-Myelin Oligodendrocyte Glycoprotein (MOG) and anti-Aquaporin-4 (AQP4) antibodies were absent, and the search for oligoclonal bands turned negative. Cerebrospinal fluid (CSF) examination demonstrated a clear rock water, marked pleocytosis (132 cells/microLiter, 99% mononuclear), and high CSF proteins (128 mg/dL). MRI of the brain and spinal cord was acquired (Figure 1, Figure 2 and Figure 3).

The integration of clinical and imaging characteristics allowed ADEM diagnosis.

Follow-up imaging revealed that the ADEM lesions were resolved and that no new lesions had appeared. As a result, the disorder was monophasic.

### 2.2. Case Presentation 2

**Figure 4:**
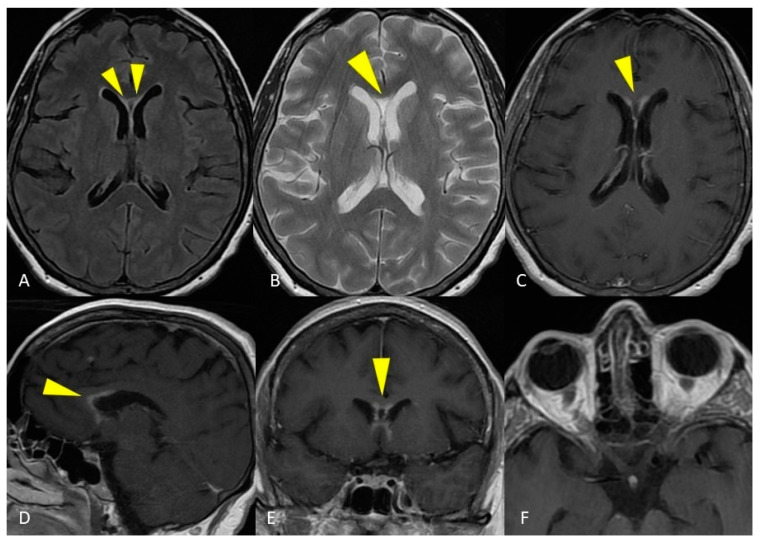
MRI of the brain. Axial T2-weighted and FLAIR images show curve hyperintense lesions affecting the ependymal surface of frontal horns of the lateral ventricles and corpus callosum just near the genu, with a symmetrical pattern (**A**,**B**). These sub-ependymal lesions show homogeneous enhancement on the T1-weighted post-gadolinium image (**C**–**E**). No optic nerve enhancement on T1-weighted post-gadolinium images was present (**F**).

**Figure 5:**
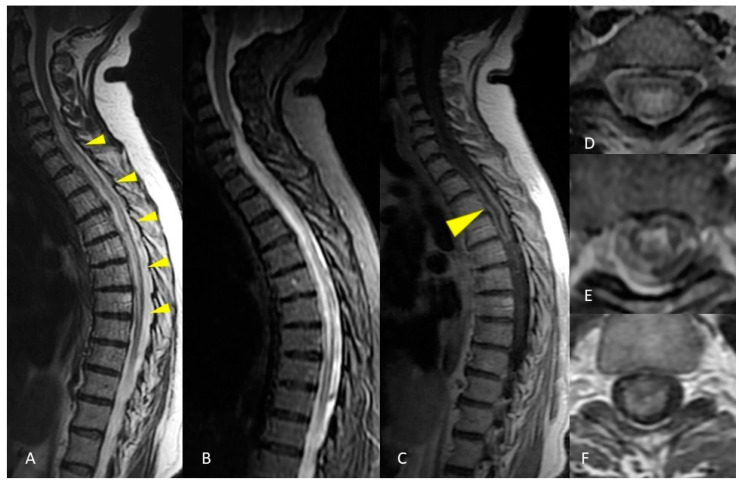
MRI of the spinal cord. Sagittal T2-weighted and STIR images show a long and continuous segment of abnormal hyperintensity affecting the cervico-thoracic spinal cord (**A**,**B**). Axial T2-weighted image shows diffuse hyperintensity involving all the cross-sectional area of the spinal cord at a more cranial level (**D**) and a predominant gray matter involvement at a more caudal level (**E**). Spinal cord swelling and enhancement were present at the segmental thoracic level, evident in both sagittal (**C**) and axial (**F**) images.

A 57-year-old female presented to the emergency department with paresthesia of the trunk, pelvis, and lower limbs with progressive difficulty of movement up to the inability to walk and collapse when standing. On the same day, double sphincter disorder was reported. The diagnosis of spinal cord syndrome was considered after neurological counseling. The patient was hospitalized for diagnostic and therapeutic evaluation. The patient was alert and cooperative, with visual acuity reduction on the left associated with retrobulbar pain. The patient presented weakness of the upper limbs and severe deficiency of the lower limbs. Osteo-tendon reflexes were normal in the upper limbs but absent in the lower limbs. Anti-MOG antibodies and oligoclonal bands were absent. Anti-AQP4 antibodies were present. Microbiological examination of CSF and peripheral blood were positive for previous and latent human beta-herpesvirus-7 (HHV 7) infection. Marker of active HHV 7 infection were excluded. MRI of brain and spinal cord was acquired (Figure 4 and Figure 5).

Despite the absence of alterations of optic nerves on MRI, which represents a major criteria for NMO diagnosis, the integration of clinical and imaging features allowed the aforementioned diagnosis.

## 3. Discussion

Autoimmune demyelinating disorders such as ADEM and NMO may be particularly challenging to distinguish from MS, hampering a prompt and accurate diagnosis [10]. MRI is currently the most valuable tool in diagnosis and differential diagnosis of ADD. However, complex radiological findings can overlap, leading to misinterpretation, confusion or misdiagnosis [11]. The two presented cases showed MRI findings that could suggest the diagnosis of MS.

### 3.1. Multiple Sclerosis

MS is an acquired inflammatory demyelinating disease of the central nervous system (CNS). MS has high prevalence in northern Europe and is rare in regions located near the equator. For unknown reasons, women are affected more frequently than men (with a 3:1 ratio) like in most diseases defined as autoimmune [12]. The typical onset of the disease is between the ages of 20–40 years manifesting with radiologically isolated syndrome (RIS), clinically isolated syndrome (CIS) and clinically definite MS [13]. Clinically definite MS can have a relapsing remitting (RRMS), primary progressive (PPMS), secondary progressive (SPMS) and progressive relapsing course (PRMS) [14]. The onset of multiple sclerosis varies on the basis of the location of lesions, but patients most commonly present with a clinically isolated syndrome (CIS), which is the first presentation of RRMS, manifesting with acute unilateral optic neuritis, incomplete myelitis or brainstem syndrome [15]. In contrast to RRMS, the PPMS is characterized by insidious onset of symptoms, usually with a slowly progressive myelopathy (most frequently asymmetric paraparesis) [16]. Diagnosis of MS is based on neurological examination to determine the presence of certain clinical symptoms and signs and is supported by other tests, such as MRI, evoked potential tests in visual, sensory, or auditory pathways and cerebrospinal fluid (CSF) analysis. MRI is highly recommended in patients with symptoms and signs suggestive of MS due to the high sensitivity to detect typical brain and spinal cord lesions [17]. MS is characterized by perivenular inflammation and demyelination, manifesting as periventricular, infratentorial, juxtacortical, and spinal cord lesions [18]. Periventricular white matter lesions are hyperintense on T2-weighted images, ovoid, perpendicular to the ventricle, with a perivenular topography (so-called “Dawson’s fingers”), and appear dark on T1-weighted images (“black holes”). The corpus callosum lesions are localized at the calloso-septal interface. Generally, they are small in size, focal, and separated from each other, determining the typical subcallosal “dot-dash” appearance. Another classic MS location is the involvement of subcortical U-fibers, as isolated juxtacortical white matter hyperintensity on T2-weighted images: this type of lesion is relatively specific for MS; on the other hand, the lesions can also be localized in the cortex [2,18]. More than 90% of patients with clinically definite MS have spinal cord abnormalities, although isolated spinal cord lesions can occur in 25% of patients [19]. The cervical region is the most commonly affected segment of the spinal cord. Typically, the lesions are short (1–2 vertebral bodies) in craniocaudal extent, often multifocal and asymmetric, and affect less than half of the cross-sectional area of the cord [1,20]. The lesions can demonstrate contrast enhancement or cord swelling in active demyelination MS [20]. According to the McDonald 2010 criteria, the diagnosis of MS requires the evidence of dissemination in time and space of demyelinating lesions, including in patients with CIS [21]. The MRI dissemination in space is defined by the presence of a T2 lesion in at least two of the four classical sites of white matter disease (juxtacortical, periventricular, infratentorial regions, and spinal cord), whereas dissemination in time requires simultaneous presence of asymptomatic gadolinium-enhancing and non-enhancing lesions, or the appearance of a new lesion during the follow-up [21].

### 3.2. Acute Disseminated Encephalomyelitis—ADEM

ADEM is a demyelinating CNS disease which mainly affects the pediatric population, with higher incidence and prevalence in regions distant from the Equator and a slight male predominance (M:F ratio up to 2.6:1) [22,23]. The incidence of the disease in the population is between 0.3–0.6 per 100,000 per year [24]. Clinical presentation of ADEM is highly variable, often preceded by a prodromal phase with fever, headache and malaise, followed by neurological alterations including brainstem symptoms, optic neuritis and transverse myelitis [25]. Moreover, all ADEM patients have encephalopathy during the acute phase of the disease with alteration in consciousness or behavior changes. The International Pediatric Multiple Sclerosis Society Group (IPMSSG) criteria, updated in 2013, recognize encephalopathy as a mandatory feature for the diagnosis of ADEM [26]. Other diagnostic features are the presence of multifocal neurological symptoms and the evidence of MRI demyelinating lesions [27]. CSF findings are non-specific, with mild pleocytosis, protein elevation in 17–48% of cases, and oligoclonal bands in 0–20% of cases [28]. ADEM has a monophasic and rapidly progressive course, but recently different subtypes are recognized, namely Multiphasic ADEM (MDEM), ADEM-Optic Neuritis (ADEMON), and acute hemorrhagic leukoencephalopathy (AHL) [29,30]. Brain lesions in ADEM are determined by perivenular inflammation leading to large areas of demyelination [31]. Typical ADEM MRI findings on T2-weighted and FLAIR images appear as bilateral, asymmetrical, multiple, confluent, poorly marginated, hyperintense areas with random distribution (leopard skin regional distribution) [32]. ADEM lesions typically involve both central white and deep grey matter, but they can also be located in the cortical gray-white matter junction, in the cerebellum and in the brainstem, sometimes with gadolinium enhancement (up to 20% of cases) or large perilesional edema, as observed in tumefactive lesions [33,34]. Unlike multiple sclerosis, lesions in ADEM do not involve the calloso-septal interface, spare the periventricular white matter and do not present with Dawson’s fingers lesions. [35,36,37]. Spinal cord myelitis is seen approximately in one-third of patients, as spinal cord lesions extended for more than two vertebral segments [38].

In the patient of case 1, the brain lesions location could lead to the misdiagnosis of MS, in particular for the presence of perpendicular periventricular lesions (Dawson’s fingers). Nevertheless, the sagittal FLAIR image showed hyperintense lesions affecting the ependymal surface of the corpus callosum with a marble pattern, as observed in NMO. On the other hand, the involvement of the spinal cord, both on axial and sagittal plane, as well as the lack of enhancement of spinal cord lesions suggested the diagnosis of ADEM. In this case, it was not possible to make an accurate diagnosis without the support of clinical and laboratory data: CSF analysis showed pleocytosis without oligoclonal bands, and the ADEM diagnosis was reasonably confirmed. This patient presented MRI brain findings atypical for ADEM diagnosis.

### 3.3. Neuromyelitis Optica—NMO

Neuromyelitis optica spectrum disorders (NMOSD) are severe autoimmune inflammatory demyelinating diseases of the CNS with frequent involvement of the optic nerves and spinal cord [39]. The NMO antibody was recognized in 2004 as an immunoglobulin-G (IgG) directed against an astrocyte water channel named Aquaporin-4 (AQP4) [40]. In AQP4-IgG seropositive patients a wide spectrum of autoimmune disorders was recognized, hence the use of the term NMO spectrum disorders (NMOSD). The incidence and prevalence of NMOSD are higher in non-Caucasians (Asians and in those of African ancestry) [41,42]. NMO have a female preponderance (F:M ratio up to 9:1) [43]. In 2015, the International Panel for NMO Diagnosis (IPND) revised the diagnostic criteria for NMOSD according to anti-AQP4 antibody status with an emphasis on six locations of the lesions (spinal cord, optic nerves, area postrema, diencephalon, brainstem and cerebrum) and typical MRI features [44,45]. Unlike MS, in NMOSD population CSF analysis usually shows a low prevalence of oligoclonal bands [46,47]. Furthermore, during the acute phase and the relapses, a variable pleocytosis consisting of neutrophils and eosinophils may be observed [48]. In literature, the characteristic MRI features of NMO are reported as confluent and asymmetrical hyperintense lesions on T2-weighted and FLAIR images in typical areas where AQP4 is consistently expressed (optic nerve, periependymal regions, structures around the third and fourth ventricles and the cerebral aqueduct, spinal cord, optic chiasm, hypothalamus, subpial areas, brainstem, and area postrema) [49]. Two of the most distinct features of NMOSD are the corticospinal tracts involvement (23–44% of patients) and the dorsal brainstem periependymal lesions involving the area postrema (7–46% of patients) [49,50]. NMOSD can also present with tumefactive hemispheric brain lesions (>3 cm) [44]. Longitudinally-extensive transverse myelitis (LETM) is the typical spinal cord feature in NMOSD, characterized by the involvement of the spinal cord with a longitudinal extension of three or more adjacent vertebrae [1,20,41,51]. The length of the spinal cord lesions in NMOSD has been considered the most important feature of differential diagnosis with MS, in which lesions are shorter than two vertebral segments [52]. Cervical, thoracic, or both spinal segments are usually compromised: cord swelling and irregular enhancement are typical of the acute phase [49]. Moreover, in NMOSD the central gray matter of the spinal cord is the area where lesions typically occur due to the higher expression of AQP4 antigen, and lesions can involve more than 50% of the spinal cord section [50]. Compared with LETM in NMOSD, myelitis in MS not only has a shorter extension but also has a more peripheral distribution, with the involvement of the spinal white matter [49]. Optic neuritis in NMOSD may differ from optic nerve involvement occurring in MS because patients usually have an early severe visual loss due to bilateral and extensive involvement of the optic nerves, extending to the optic chiasm, with a poor response to corticosteroid therapy and a frequent relapsing course, whereas optic neuritis in MS is usually unilateral, with a shorter involvement of the optic nerve and infrequent extension to the optic chiasm [39,53].

In the patient of case 2, the main laboratory finding was the presence of positive serum and CSF anti-AQP4 antibodies, without oligoclonal bands. This finding suggested the diagnosis of NMO. However, MRI reported the absence of visible signs of optic neuritis, a major criteria for NMO diagnosis [54]. On the other hand, the NMO diagnosis was supported by the ependymal surface location of brain lesions, the involvement of a very long and continuous segment of the cervico-thoracic spinal cord and the involvement of all the cross-sectional areas of the spinal cord [50,55].

### 3.4. Idiopathic Transverse Myelitis

Namely, Transverse Myelitis (TM) is an acute or subacute inflammation of the spinal cord, which can be idiopathic (iTM) or secondary to other diseases [56]. Incidence of iTM is of 1.34–4.6 per million per year, with a distribution in the second and fourth decade, no gender prevalence, and most commonly involving the thoracic tract [57,58,59,60]. TM symptoms progress in hours or a few weeks, and mostly include back pain, paresthesies, paraparesis, bladder symptoms and sensory level. Spine MRI shows abnormal cord signal [61,62,63,64]. CSF examination shows pleocytosis and high protein levels. iTM usually presents with oligoclonal bands in CSF (62%) [58]. As TM can be a prelude of MS, findings of disease progression must not be overlooked [58,65,66,67,68,69,70]. Cord lesions in acute iTM are usually central, circumferential, uniform and symmetric in comparison to MS which typically has patchy and peripheral lesions [71]. Literature findings of auto-antibodies in TM are reported, as well as of Interleukin-6 or IgE-mediated damage [72,73,74,75,76,77,78,79,80].

### 3.5. Myelin Oligodendrocyte Glycoprotein Encephalomyelitis

MOG antibody disorders typically occur in young Caucasian patients, with low female prevalence [33,81,82,83,84,85]. Clinically, MOG antibody disease that involves the optic nerve and spinal cord resembles NMOSD, with sight loss and paresis, and in case of brain involvement it has a presentation similar to ADEM, with encephalitis [82,86,87]. However, unlike anti-AQP4 antibodies which attack the astrocytic AQP4 protein, anti-MOG antibodies attack a protein expressed on the outer surface of the myelin, leading to greatly demyelinating episodes than NMOSD; therefore, on the immunopathological side it is closer to MS [82,88]. Literature evidence seems to suggest that the ADEM-like encephalitic presentation of MOG disease, with headache, mental status change, seizures, and neurological deficits depending on lesion location, is more frequent in younger patients [89,90]. MOG disease usually has a monophasic course, even though literature evidence also reports a relapsing course [91,92]. CSF findings in MOG disease are characterized by pleocytosis with lymphocytes and rare oligoclonal bands (20.25%) [89,90]. MRI findings in MOG disease presenting with encephalitis show an ADEM-like pattern, with subcortical and deep white matter as well as grey matter lesions in T2-weighted and FLAIR sequences, sometimes rendering these two entities radiologically indistinguishable; in some cases, lesions can demonstrate restricted diffusion [89,90]. MOG disease presentation and MRI findings can be severe, but usually with better outcomes than NMOSD and complete resolution on MRI follow-up [91,92]. Diagnostic criteria for MOG encephalomyelitis include serum and/or CSF MOG-IgG positivity, any neurological disease (including ADEM, NMO, transverse myelitis, brain or brainstem syndromes), and absence of alternative diagnoses [93].

### 3.6. CLIPPERS

Chronic lymphocytic inflammation with pontine perivascular enhancement responsive to steroids (CLIPPERS) is a rare inflammatory CNS disorder involving predominantly the brainstem, described in 2010 for the first time by Pittock and colleagues [94]. Although the pathogenesis of CLIPPERS is still not clearly known, the neuropathologic examination demonstrates perivascular chronic lymphocytic inflammation involving predominantly the white matter of the pons [95]. The hallmark of CLIPPERS is the excellent clinical and radiologic response to corticosteroid treatment [96]. Clinical symptoms are various and related to brainstem, cerebellar and cranial nerve involvement (ataxia, diplopia, dysarthria, diplopia and vertigo are the most frequent), frequently accompanied by other symptoms as myelopathy and cognitive dysfunctions [96]. Despite there are no available diagnostic criteria, diagnosis of CLIPPERS is currently based on core findings including clinical, radiological and histopathological ones, and laboratory and CSF evaluation in order to exclude alternative diagnosis [97]. Specific diseases included in differential diagnoses of CLIPPERS are CNS infections, CNS lymphoma, intravascular lymphoma, vasculitis, and CNS demyelinating disease such as MS, ADEM, and NMO [98]. Brain MRI in CLIPPERS has a characteristic punctate and/or curvilinear pattern of post-gadolinium enhancement in the pons, that tend to correspond to the areas of T2-weighted and FLAIR hyperintensity, with or without extension into the cerebellar peduncles, the medulla, the midbrain and the cerebellum [99]. In some patients, these enhancing lesions were found in the spinal cord and in supratentorial CNS regions (e.g., basal ganglia, capsula interna, thalami, corpus callosum, and hemispheric white matter) [99]. In CLIPPERS there are no tumefactive lesions, exceptional cases excluded, and the enhancement usually decreases with corticosteroid therapy [100].

## 4. Conclusions

The two cases presented in this paper highlight well the challenges that need to be solved in order to formulate an accurate diagnosis: the presence in both patients of hybrid MRI findings that could be present in more than one ADD, could have easily led to an incorrect interpretation and misdiagnosis. This does not mean that, in this scenario, the role of MRI has weakened, rather that the integration of MRI findings with clinical and laboratory data is mandatory. These cases and the literature review underline the complexity of magnetic resonance imaging in autoimmune demyelinating diseases diagnosis and the crucial need of clinical and laboratory data integration. Some magnetic resonance findings can interchange between different demyelinating pathologies or can not be expressed simultaneously in a single demyelinating disease.

## Data Availability

Not applicable.
